# Case Report of a Pressure Ulcer Occurring Over the Nasal Bridge Due to a Non-Invasive Ventilation Facial Mask

**DOI:** 10.7759/cureus.813

**Published:** 2016-10-03

**Authors:** Farooq A Rathore, Faria Ahmad, Muhammad Umar U Zahoor

**Affiliations:** 1 Department of Rehabilitation Medicine, PNS Shifa Hospital, DHA II, Karachi 75500, Pakistan; 2 Department of Rehabilitation Medicine, Bahria University Medical and Dental College, Bahria University, DHA -II, Karachi; 3 Department of Rehabilitation Medicine, Faculty of Rehabilitation Medicine, University of Alberta, Edmonton, Canada; 4 Department of Rehabilitation Medicine, CMH Lahore Medical College and Institute of Dentistry, Lahore, Pakistan; 5 Anesthesia and Pain Medicine, CMH Lahore Medical College and Institute of Dentistry, Lahore, Pakistan

**Keywords:** pressure ulcer, intensive care, ventilation, wounds, prevention, nursing

## Abstract

Non-invasive ventilation (NIV) is used in patients with respiratory failure, sleep apnoea, and dyspnoea related to pulmonary oedema. NIV is provided through a facial mask. Many complications of NIV facial masks have been reported, including the breakdown of facial skin. We report a case of an elderly male admitted with multiple co-morbidities. The facial mask was applied continuously for NIV, without any relief or formal monitoring of the underlying skin. It resulted in a Grade II pressure ulcer. We discuss the possible mechanism and offer advice for prevention of such device-related pressure ulcers.

## Introduction

Non-invasive ventilation (NIV) is indicated in patients with respiratory failure, sleep apnoea, and dyspnoea related to pulmonary oedema [1]. The face mask needs to be tight around the nose and mouth in order to create an effective seal. NIV reduces the need for invasive ventilation, reducing related morbidity and mortality. It also reduces the additional costs associated with invasive ventilation.

However, NIV has its own limitations and potential complications. Pressure-related skin and tissue necrosis is one of them. The most important cause is the pressure above the normal capillary filling pressure of the skin, along with contributing factors such as poor hydration, hypotension, and skin thinness due to anatomy and use of corticosteroids. Pressure-induced ischemia at 35 mm of Hg for a duration of two hours is enough to induce tissue damage and necrosis [2]. Therefore, guidelines suggest a continuous application of continuous positive airway pressure (CPAP) for six to eight hours with intermittent breaks to ensure that the patient receives the required amount of oxygen [3].

The authors present a case report of an unusual pressure ulcer developing in an uncommon location (bridge of the nose) due to the prolonged application of an NIV facial mask.

## Case presentation

**Figure 1:**
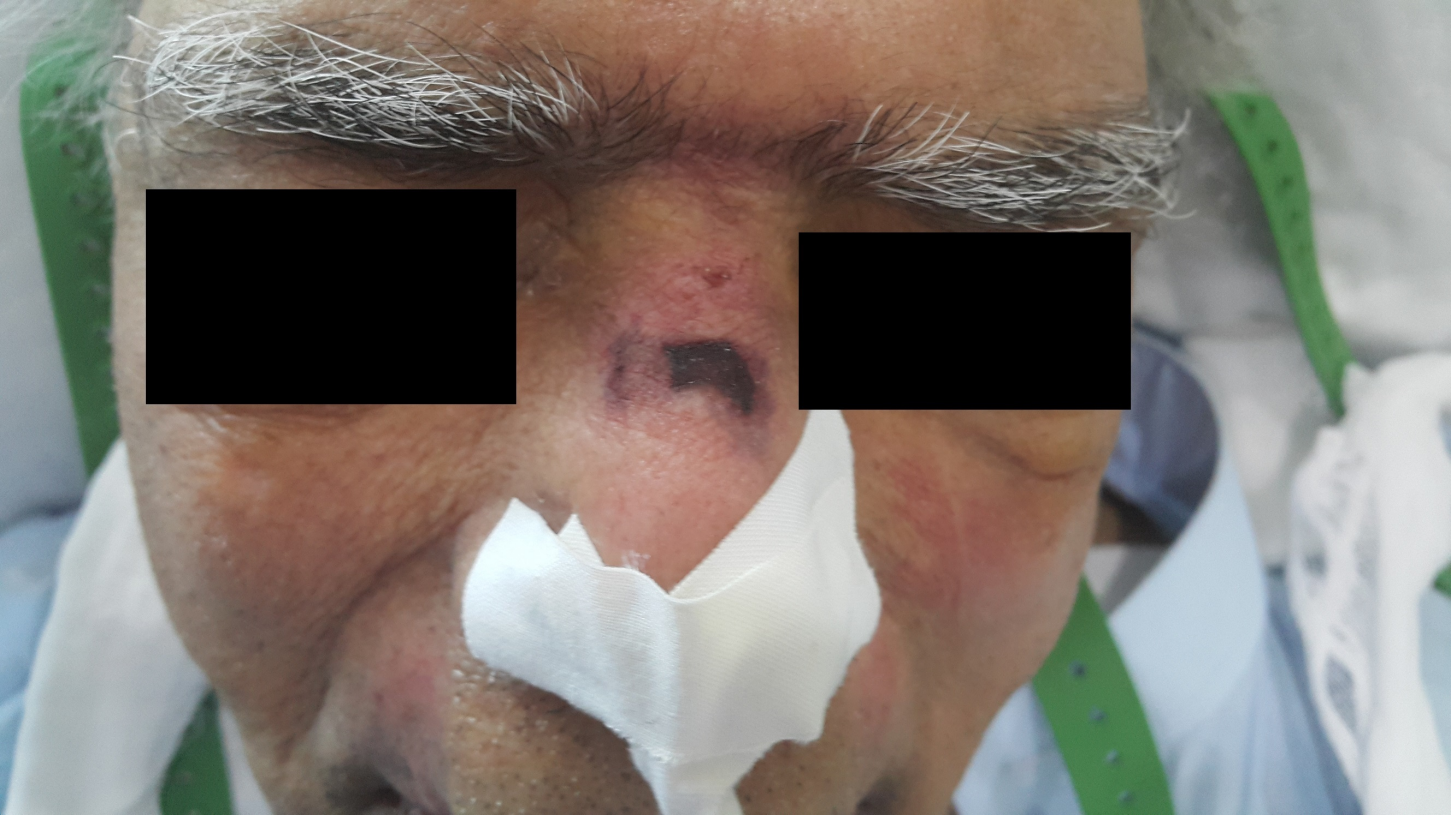
Grade II pressure ulcer on the bridge of the nose

An 86-year-old man presented to the emergency department with chronic respiratory failure and severe pneumonia. Comorbidities included diabetes, uncontrolled hypertension, ischemic heart disease, Parkinson’s disease, and bronchial asthma. As his condition continued to deteriorate further, he was admitted to the intensive care unit (ICU). The patient was unable to maintain adequate arterial oxygen saturation via a face mask and nasal prongs. The attending physician ordered non-invasive ventilation (NIV) and a continuous positive airway pressure (CPAP) mask was applied. On day three of his ICU stay, one of the authors noticed a Grade II pressure ulcer on the bridge of his nose (Figure 1).

Examination of the patient showed a tight-fitting full facial mask. On inquiry, the caregivers revealed that the CPAP mask had been kept in place for almost 24 hours without any formal monitoring or inspection of pressure areas. The patient’s family refused to consent to the physician's request for invasive ventilation. Unfortunately, the patient progressively deteriorated due to worsening of his septicemia and respiratory failure and died five days following his admission to ICU. Verbal informed consent to present the case report was obtained from the immediate family members.

## Discussion

The NIV is primarily being used to improve oxygenation in patients with chronic obstructive pulmonary disease and pulmonary oedema [4]. It is also used for patients with obstructive sleep apnea at home. As these patients require the device for longer periods of time, complications  related to NIV are common [5]. They may include hypotension, barotrauma, acute cardiogenic pulmonary oedema, CO2 rebreathing, claustrophobia, patient–ventilator dyssynchrony, underlying skin irritation, skin breakdown, and the risk of aspiration [6]. Our patient's comorbidities included Parkinson’s disease, and he had been admitted to hospital with a diagnosis of bilateral lower lobe pneumonia and respiratory distress. With many underlying comorbidities, it was therefore decided to maintain the patient on a trial of bilevel positive airway pressure (BiPAP) for as long as necessary. The pressure ulcer developed overnight as the facial mask was not removed due to the patient’s respiratory condition and probably due to a lack of vigilance on the part of the nursing staff. The mask was fastened tightly to maintain positive airway pressure and oxygenation. Later on, when the pressure ulcer on the bridge of the nose was detected, the mask was used intermittently (two hours on, 30 mins off ). However, the patient died due to pulmonary complications five days following admission.

Although facial skin breakdown and pressure ulcers over the bridge of the nose due to facial masks for NIV have been reported, they continue to occur [4, 6-7]. The aim of this case study is to highlight the early identification and prevention of pressure ulcers associated with NIV. The tight seal of the face mask, which has to be maintained for long periods, is the source of discomfort, pain, and feeling of suffocation for many who use NIV [6]. These reasons account for the high failure rate of patient compliance [8]. One contributing factor for this discomfort is the use of an inappropriately sized device. Patients with marked nasal anatomy are also at higher risk of developing pressure ulcers over the nasal bridge, especially with corticosteroid use, which plays a part in thinning the skin and, thus, makes the nasal bridge susceptible to easy bruising and injury [9]. Therefore, it is important that the mask has a larger cushion size, spreading the contact pressure over a larger skin area and minimizing the shear forces on the nose during inspiration and expiration [10]. Attention is required to develop a device made from soft, malleable material to facilitate customized usage to correctly fit over the individual’s nose and mouth.

Prevention strategies may include the use of thin hydrocolloids, film dressings, or barrier products underneath the device to reduce moisture, friction, and shear. In addition, pressure-reducing dermal gel pads can be used. In developing countries, such as Pakistan, these resources are not available in the majority of hospitals. To provide comfort and prevent the development of pressure ulcers, alternatives such as surgical gauze, tissue paper, or cotton soaked in lignocaine gel can be used.

In this case, the pressure ulcer formed over the nasal bridge due to the continuous application of a CPAP face mask for a consecutive three-day period. In order to prevent further progression of the ulceration, gauze pieces were placed under the facemask seal and skin

It is imperative that nursing staff maintain adequate documentation relating to the time interval for NIV usage on all patients in their care and properly communicate this fact to the next team of nurses coming on duty. Special attention should be paid to developing oedema under the device due to prolonged pressure or due to administration of IV fluids, which may increase ulceration and delay healing. Ultimately, evidence-based practice and continuing education for all healthcare professionals should be provided in order to create awareness and to reduce the risk of this complication.

## Conclusions

NIV is routinely used in hospitals. The use of NIV can be associated with a number of complications, including pressure ulcers. Prevention, vigilance, and early identification of pressure ulcers is the key to increasing patient compliance and reducing morbidity.
